# Cerebral venous sinus thrombosis in traumatic brain injury: A systematic review of its complications, effect on mortality, diagnostic and therapeutic management, and follow-up

**DOI:** 10.3389/fneur.2022.1079579

**Published:** 2023-01-09

**Authors:** Dag Ferner Netteland, Else Charlotte Sandset, Magnus Mejlænder-Evjensvold, Mads Aarhus, Elisabeth Jeppesen, Diana Aguiar de Sousa, Eirik Helseth, Tor Brommeland

**Affiliations:** ^1^Department of Neurosurgery, Oslo University Hospital Ullevål, Oslo, Norway; ^2^Faculty of Medicine, University of Oslo, Oslo, Norway; ^3^Department of Neurology, Oslo University Hospital Ullevål, Oslo, Norway; ^4^Department of Research, Norwegian Air Ambulance Foundation, Oslo, Norway; ^5^Department of Radiology and Nuclear Medicine, Oslo University Hospital Ullevål, Oslo, Norway; ^6^Faculty of Health Studies, VID Specialized University, Oslo, Norway; ^7^Department of Neurosciences (Neurology), Hospital de Santa Maria, University of Lisbon, Lisbon, Portugal

**Keywords:** cerebral venous sinus thrombosis, traumatic brain injury, complications, mortality, diagnosis, management, follow-up, systematic review

## Abstract

**Objective:**

Cerebral venous sinus thrombosis (CVST) is increasingly being recognized in the setting of traumatic brain injury (TBI), but its effect on TBI patients and its management remains uncertain. Here, we systematically review the currently available evidence on the complications, effect on mortality and the diagnostic and therapeutic management and follow-up of CVST in the setting of TBI.

**Methods:**

Key clinical questions were posed and used to define the scope of the review within the following topics of complications; effect on mortality; diagnostics; therapeutics; recanalization and follow-up of CVST in TBI. We searched relevant databases using a structured search strategy. We screened identified records according to eligibility criteria and for information regarding the posed key clinical questions within the defined topics of the review.

**Results:**

From 679 identified records, 21 studies met the eligibility criteria and were included, all of which were observational in nature. Data was deemed insufficiently homogenous to perform meta-analysis and was narratively synthesized. Reported rates of venous infarctions ranged between 7 and 38%. One large registry study reported increased in-hospital mortality in CVSP and TBI compared to a control group with TBI alone in adjusted analyses. Another two studies found midline CVST to be associated with increased risk of mortality in adjusted analyses. Direct data to inform the optimum diagnostic and therapeutic management of the condition was limited, but some data on the safety, and effect of anticoagulation treatment of CVST in TBI was identified. Systematic data on recanalization rates to guide follow-up was also limited, and reported complete recanalization rates ranged between 41 and 86%. In the context of the identified data, we discuss the diagnostic and therapeutic management and follow-up of the condition.

**Conclusion:**

Currently, the available evidence is insufficient for evidence-based treatment of CVST in the setting of TBI. However, there are clear indications in the presently available literature that CVST in TBI is associated with complications and increased mortality, and this indicates that management options for the condition must be considered. Further studies are needed to confirm the effects of CVST on TBI patients and to provide evidence to support management decisions.

**Systematic review registration:**

https://www.crd.york.ac.uk/prospero/, identifier: PROSPERO [CRD42021247833].

## Introduction

Cerebral venous sinus thrombosis (CVST) refers to clot formation in the dural venous sinuses. The obstruction of venous outflow may result in complications in the form of brain edema, venous infarction, and hemorrhage in the drainage area of the affected dural venous sinus (1, 2). In addition, CVST, especially of the superior sagittal sinus, may cause increased intracranial pressure by disrupting circulation and absorption of cerebrospinal fluid (1, 2). All-cause CVST most often affects young adults and children and is associated with a mortality rate of up to 8%, but most often a good neurological outcome among survivors (3).

CVST associated with traumatic brain injury (TBI) is a specific subtype of the condition. Contrary to spontaneous CVST, it is typically caused by a direct mechanical disruption of the integrity of the vessel wall, and fractures and epidural hematomas overlying a dural venous sinus are recognized risk factors for its development (4–7). As advanced imaging is becoming more readily available, cerebral venous sinus thrombosis (CVST) is increasingly being recognized in the setting of TBI. In a recently published systematic review and meta-analysis evaluating the prevalence of CVST in TBI (8), a pooled frequency of CVST of 26.2% was found among TBI patients with skull fractures adjacent to a cerebral venous sinus. However, the effect of CVST on TBI patients is not well-established, although several recent studies have indicated that CVST is associated with increased risk of mortality (9–11). These indications that CVST may not be uncommon and not necessarily benign in the setting of TBI, lead to challenges in the clinical setting. Although management guidelines exist for spontaneous CSVT (12), the optimal management and follow-up of the condition in the setting of concurrent TBI remains undetermined, and specific guidelines are non-existent.

Here, we systematically review the available evidence on the complications, effect on mortality and the diagnostic and therapeutic management and follow-up of CVST in TBI. Furthermore, we discuss the management and follow-up of the condition in the context of the available evidence.

## Methods

The scope of this review was defined by key clinical questions formulated in the population, intervention, comparator/control, and outcomes (PICO) format (13) (Table 1). The key clinical questions were grouped into the following topics: complications; effect on mortality; diagnostics; therapeutics; recanalization; and follow-up. The order of the topics was based on the logic that complications from CVST and its effect on mortality in TBI patients form a basis on which the diagnostic and therapeutic management of the condition should be considered. For the purpose of this review, complications from CVST was defined as venous infarction, intracerebral hemorrhage and focal or generalized edema secondary to CVST. Recanalization and follow-up were grouped together based on the logic that recanalization informs how the condition should be followed. A research protocol was developed and registered in the international prospective register of systematic reviews (PROSPERO), in accordance with PRISMA guidelines (14) (PROSPERO CRD42021247833).

**Table 1:** Key clinical research questions (PICO format).

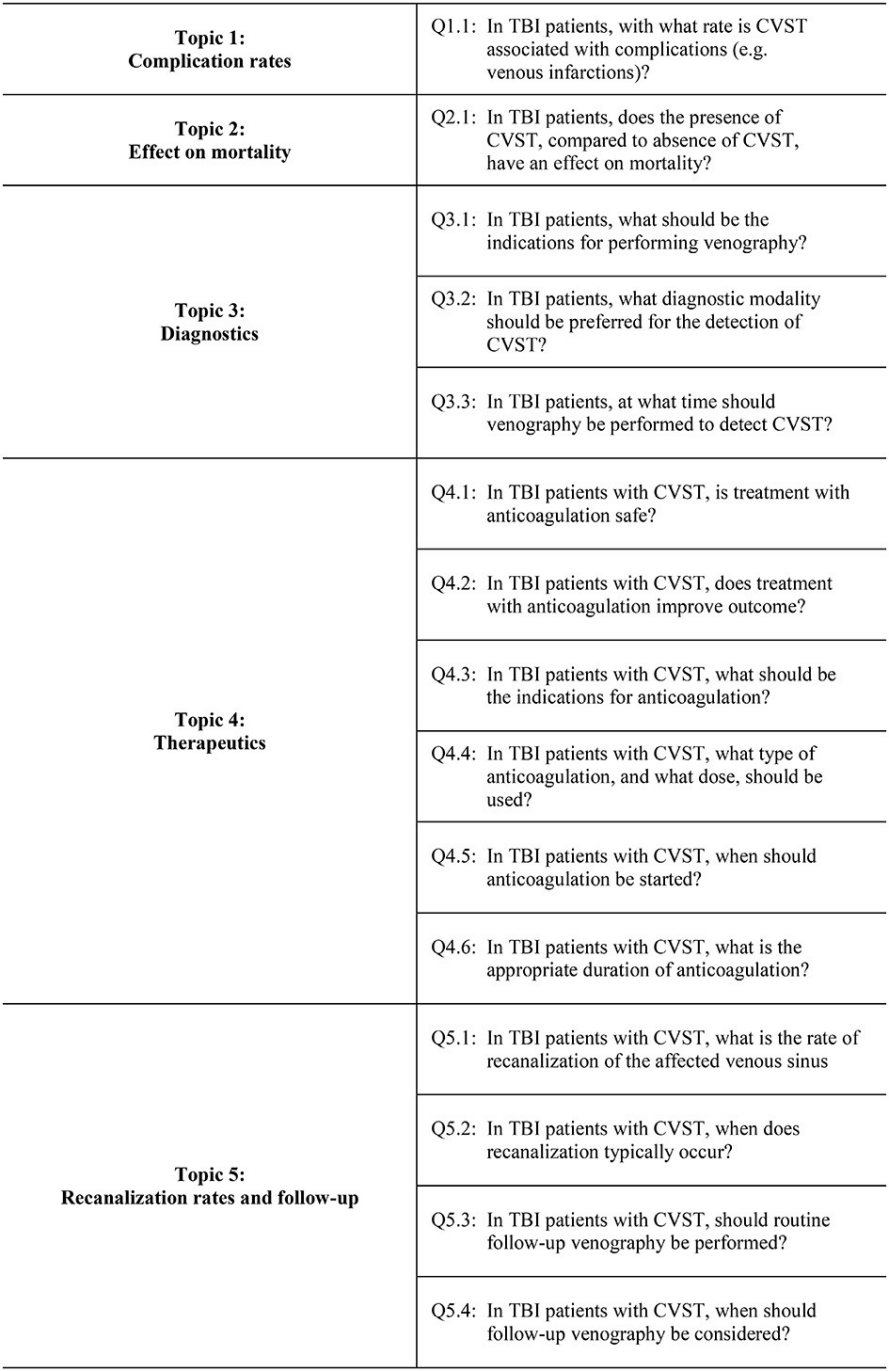

### Search strategy

The literature searches were conducted by two librarians and the search strategy was recorded. The following terms and combinations thereof were used: traumatic, trauma, cerebral, cranial, intracranial, venous, venous sinus, dural venous sinus, sinus-vein, sinovenous, thrombosis, occlusion, injury, management, treatment, anticoagulation. The following databases were searched: Pubmed, MEDLINE, Embase, The Cochrane Library, Epistemonikos and relevant sources of clinical guidelines [Brain Trauma Foundation, BMJ best practice, National Institute for Health and Care Excellence (NICE)]. Selected reference lists of retrieved studies were searched to further identify potentially eligible publications. The first systematic search was conducted 16 Oct 2020. The search was updated to include end of March 2022.

### Eligibility criteria and screening process

Criteria for study inclusion were published clinical trials or observational studies reporting CVST in patients hospitalized for TBI and providing original patient data concerning the formulated key clinical questions. Excluded were case reports, case series with less than five subjects with CVST and TBI and studies published in languages other than English.

Two authors (DFN and TB) independently first screened titles and abstracts and then full-text articles for eligibility. Any discrepancies between the two authors (DFN and TB) were resolved by discussion, first between the two authors (DFN and TB), and if necessary by discussion among all authors of the review.

### Extraction and synthesis of data and quality assessment

Full text review and data extraction from included studies was divided amongst all reviewers in pairs, where each reviewer in a pair independently reviewed allocated studies and extracted data according to the PICO questions. Each reviewer completed a standardized form providing a summary of their allocated studies which was subsequently shared with the study group. Extracted information included available data pertinent to each key clinical question. In addition, information on study-type, demographics including age group (all ages/pediatric only/adult only) and type and number of subjects with different types of cerebral venous sinus pathology was extracted. Missing data were sought by contacting study investigators enquiring about unreported data in select instances.

To synthesize the data, we grouped extracted data according to the defined topics: complications, effect on mortality, diagnostics, therapeutics, and recanalization/follow-up. Within each topic, relevant and uniformly reported data between the studies was sought and recorded. Data was narratively synthesized and summarized in table form. If deemed meaningful, data was planned to be synthesized by meta-analysis.

Quality assessment of included studies was performed using the Newcastle-Ottawa Scale for critical appraisal of observational studies (15).

## Results

**Figure 1:**
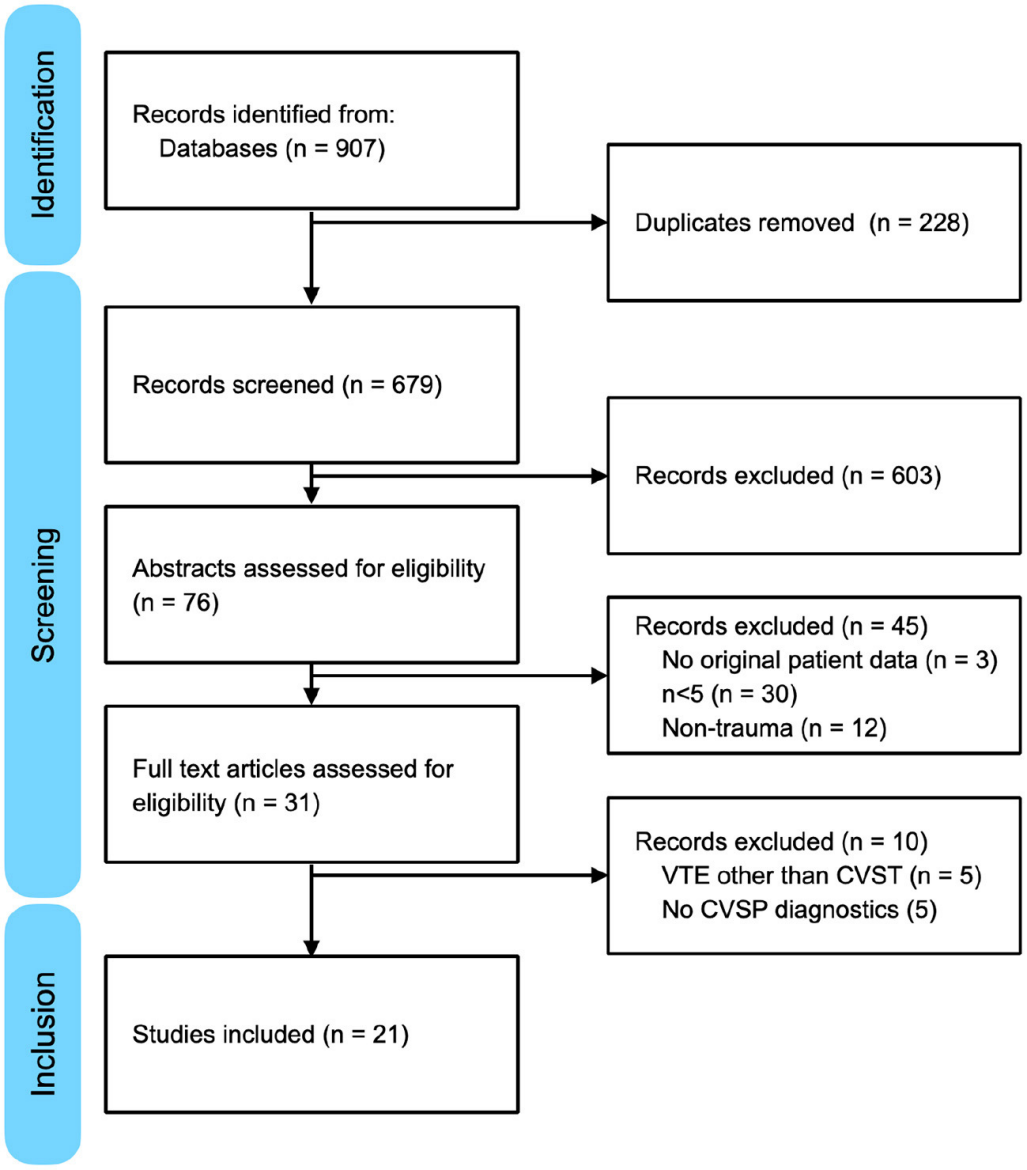
PRISMA flow diagram detailing identification, screening and inclusion of studies.

The search identified 607 records. Thirtyone full-text articles were retrieved after the exclusion of 576 records on the basis of their titles and abstracts (Figure 1). After screening of the 31 retrieved full-text articles, a total of 21 studies meeting the eligibility criteria were included in this systematic review (Table 2). All included studies were observational in nature. Four studies were series of pediatric patients < 18 years old, while the remaining 17 studies were either series of adult patients ≥ 18 years old or series of patients of all ages. Risk of bias according to the Newcastle-Ottawa scale is shown in Table 3.

**Table 2:** Overview of included studies.

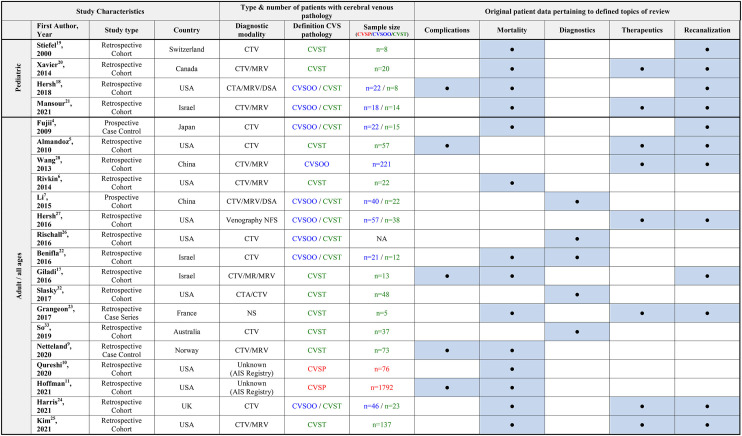

CVS, cerebral venous pathology; CVSP, cerebral venous sinus pathology; CVSOO, cerebral venous sinus outflow obstruction; CVST, cerebral venous sinus thrombosis; CTV, computed tomography venography; CTA, computed tomography angiography; MRV, magnetic resonance venography; DSA, digital subtraction angiography; AIS, abbreviated injury scale; NFS, not further specified. Red text color annotates CVSP; blue text color annotates CVSP; green text color annotates CVST.

**Table 3:** Newcastle-Ottawa scale scores for included observational studies.

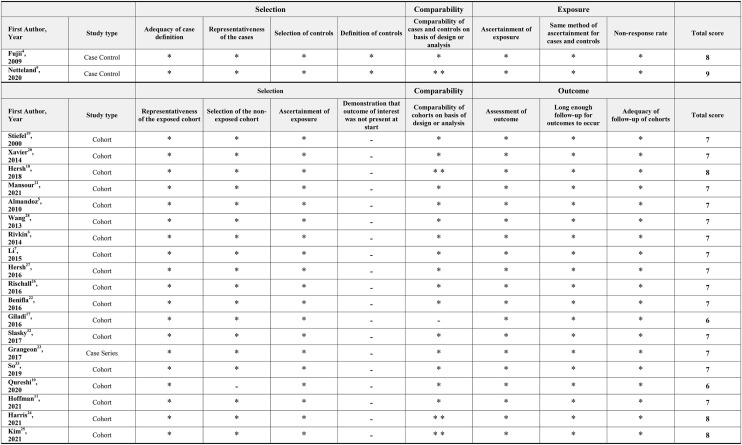

The definition of cerebral venous pathology varied between the studies. Therefore, for the purpose of this review, we chose to categorize the definitions used in the included studies into cerebral venous sinus pathology (CVSP) not further specified, cerebral venous sinus outflow obstruction (CVSOO), cerebral venous sinus thrombosis (CVST), and external compression of a venous sinus. Studies using registry data, e.g., from abbreviated injury scale (AIS) (16) based trauma registries, that include a range of codes for different sinus injuries and pathologies were categorized as CVSP. CVSOO includes both CVST and external compression of a cerebral venous sinus. CVST was defined as intramural thrombus of a cerebral venous sinus. External compression should be understood as sole external compression of a cerebral venous sinus from e.g., an epidural hematoma or a depressed skull fracture, and does not entail intramural pathology, although external compression may coexist with CVST.

A subset of studies reported complication rates and mortality data by the location of CVST, but categorization of CVST location varied between studies. When location was reported as superior sagittal sinus, torcula/confluence of sinuses or deep cerebral veins we chose to group these locations into midline location.

### Complications (topic 1)

*(Q1.1 In TBI patients, with what rate is CVST associated with complications (e.g. venous infarctions)?)*

#### Venous infarctions

Venous infarction rates were reported by three studies of patients of all ages (5, 9, 17) and one study of pediatric patients only (18). In the all-age studies the reported venous infarction rates ranged between 7 and 18%. In the single available pediatric series of a total of 8 patients with CVST, 38% (3/8) were reported to develop venous infarction.

In addition, a large registry-based study of 1,792 adult patients with CVSP found that sinus injury was associated with in-hospital stroke of any type, but the registry data used did not distinguishing between arterial and venous stroke (11). Table 4 summarizes the reported venous infarction rates.

**Table 4:** Venous infarction and in-hospital mortality rates.

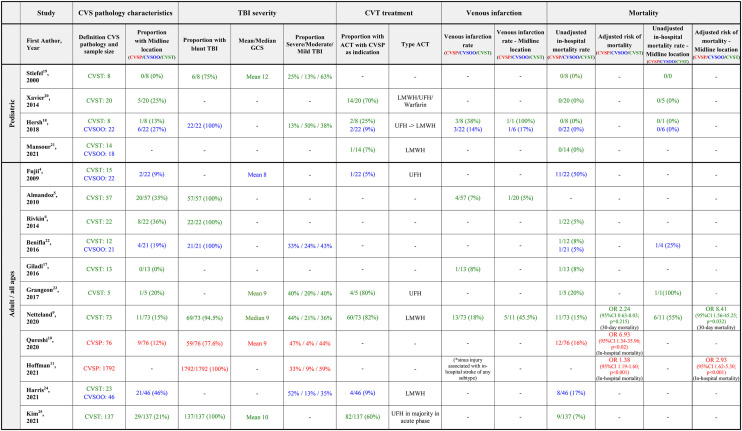

CVS, cerebral venous pathology; CVSP, cerebral venous sinus pathology; CVSOO, cerebral venous sinus outflow obstruction; CVST, cerebral venous sinus thrombosis; TBI, traumatic brain injury; Midline location, superior sagittal sinus/torcula/deep cerebral veins; Severe TBI, GCS 3–8; Moderate TBI, GCS 9–12; Mild TBI is GCS 13–15; AIS, abbreviated injury scale; ACT, anticoagulation treatment; LMWH, low molecular weight heparin; UFH, unfractionated heparin; OR, odds ratio. Red text color annotates CVSP; blue text color annotates CVSP; green text color annotates CVST.

For the subgroup of midline CVST, data was available from two all-age studies (5, 9) and one pediatric study (18). In the two all-age studies, the reported venous infarction rates for midline CVST was 5 and 46%. In the single pediatric study, only one of eight patients had midline CVST and this patient was also reported to have developed venous infarction.

#### Other complications

Only one study systematically reporting rates of complications other than venous infarctions was available (9). In this study by Netteland et al. 11% of patients with CVST were reported to have developed intracerebral hemorrhage secondary to CVST, and 16 and 3% developed focal and generalized edema secondary to CVST, respectively. Rates of complications were also found to be dependent on CVST location, with midline location being associated with significantly higher rates of complications than bilateral and unilateral CVST.

### Effect on mortality (topic 2)

*(Q2.1: In TBI patients, does the presence of CVST, compared to absence of CVST, have an effect on mortality?)*

Adjusted mortality rates were available from three studies of all-age/adult populations (9–11), while unadjusted mortality rates were reported by a number of the included studies. In-hospital mortality was the most consistently reported mortality rate in 13 of the included studies (4, 6, 9, 10, 17–25), while mortality data at set time intervals (e.g., 30-day mortality) and functional outcome measures (e.g., Glasgow outcome scale) were less consistently reported between studies.

#### Adjusted risk of mortality

Adjusted risk of mortality in groups of patients with CVST and TBI, compared to in groups of patients with TBI alone, were available from three of the included studies (9–11). All three studies included either adult patients only or patients of all ages, hence no adjusted mortality data was available from pediatric only populations.

Netteland et al. found a tendency toward, but not a statistically significant increased risk of 30-day mortality associated with CVST (OR 2.24, 95% CI 0.63–8.03, *p* = 0.215) (9). In this case-control study of 76 cases with TBI and venography confirmed CVST and 120 controls with TBI and venography confirmed absence of CVST, regression analysis was adjusted for differences in Glasgow coma scale (GCS), Rotterdam CT score and Injury severity score (ISS) between groups. Demographics, including age, were well-matched between groups.

Qureshi et al. found a significant association between CVSP and risk of in-hospital mortality [OR 6.93 (95% CI 1.34–35.96, *p* < 0.02)] (10). In this study based on US national trauma bank data, 76 patients with CVSP were identified by using abbreviated injury scale (AIS) coding from a total cohort of 453,775 patients. Of note, the total cohort consisted of patients not only with ICD coding for TBI, but also patients with ICD coding for head and neck injury in this study. Analysis was adjusted for age, gender GCS, ISS, and presence of intracranial hemorrhage.

In another study also based on national trauma bank data, Hoffman et al. found a significant association between CVSP and risk of in-hospital mortality [OR 1.38 (95% CI 1.19–1.60, *p* < 0.001)] (11). In this study, 1,792 patients with CVSP were identified using abbreviated injury scale (AIS) coding for sinus injury from a total cohort of 619,659 patients with blunt TBI. In contrast to the previously mentioned study, the total cohort here consisted of TBI patients only identified by AIS head region severity score of 3–5 and penetrating injuries were excluded. Analysis was adjusted for age, sex, race, number of comorbidities, injury mechanism, hospital teaching status, hypotension, GCS, ISS, and intracranial injury type.

For the subgroup of midline CVSP, adjusted mortality rates were available from two studies (9, 11).

In the study by Netteland et al., subgroup analysis by CVST location showed midline/bilateral CVST (*n* = 14) to be significantly associated with increased risk of 30-day mortality [OR 8.41 (95% CI 0.63–8.03, *p* = 0.215)] (9).

In the study by Hoffman et al., superior sagittal sinus CVSP was found to be significantly associated with increased risk of in-hospital mortality [OR 2.93 (95% CI 1.62–5.30, *p* < 0.001)] (11). The total number of patients with CVSP in this series was 1,792, however the number of patients with superior sagittal sinus CVSP was not reported.

Notably, both of these studies found a considerable higher risk of mortality associated with midline venous sinus pathology as compared to venous sinus pathology of any location.

#### Unadjusted mortality rates

Unadjusted in-hospital mortality rates were reported by nine adult/all-age studies (4, 6, 9, 10, 17, 22–25) and four pediatric studies (18–21). In the adult/all-age studies, in-hospital mortality rates ranged between 5 and 20% for studies where venous pathology was defined as CVST and between 5 and 50% for studies where venous pathology was defined as CVSP. For the pediatric populations, all four studies reported a 0% in-hospital mortality rate from a total of 50 patients with CVST.

TBI severity was reported in a variable manner which precluded meaningful comparison of TBI severity between studies, and therefore meaningful meta-analysis of the effects of CVST on mortality. Table 4 summarizes the reported in-hospital mortality rates.

For the subgroup of midline CVST, three all-age series (9, 22, 23) and two pediatric studies (18, 20) reported unadjusted in-hospital mortality rates for midline CVSP in TBI. In the adult/all age series, two studies using CVST as the definition of venous sinus pathology reported in-hospital mortality rates of 55% (6/11) (9) and 100% (1/1) (23) for midline CVST. Another study using CVSOO as definition for venous sinus pathology reported an in-hospital mortality rate of 25% (1/4) for midline CVSOO (22). For the pediatric populations, both studies reported a mortality rate of 0% from a total of six patients with midline CVST (18, 20).

### Diagnostics (topic 3)

#### Indications for venography

*(Q3.1: In TBI patients, what should be the indications for performing venography?)*

Regarding indications for performing venography, we identified no studies reporting systematic data to directly inform the appropriate indications for venography.

While not directly related to the key clinical questions defining the scope of this review, Li et al. found that both fractures (OR 8.03, 95% CI 3.11–20.73, *p* = 0.000) and EDH [OR 3.06 (95% CI 1.36–6.921, *p* = 0.007)] crossing a dural venous sinus were risk factors for CVST in a prospective cohort study of 240 consecutive moderate and severe TBI patients (7). Furthermore, in a recent meta-analysis, we note that Bokhari et al. found a 26.2% pooled frequency of CVST among patients with a fracture adjacent to a venous sinus compared to a pooled frequency of CVST of 4% in all TBI (8).

#### Diagnostic modality

*(Q3.2 In TBI patients, what diagnostic modality should be preferred for the detection of CVST?)*

Regarding diagnostic modality, no studies reporting comparative data between different venography modalities in the setting of TBI were identified. The vast majority of the included studies reported using CTV as diagnostic modality, either exclusively, or with MRV as an alternative. In some instances, preference to MRV was reported in pediatric cases to avoid radiation.

Benifla et al. evaluated the use of non-contrast head CT in the detection of CVST in the setting of TBI, and found this to have a poor (38%) sensitivity in detecting CTV defined CVST, even though several signs on NCCT have been associated with CVST (22).

Rischall et al. evaluated CTV interobserver reliability in the setting of TBI and found a strong interobserver reliability among three neuroradiologists (κ = 0.627–0.772; *p* < 0.0001) (26). The most common source of disagreement reported was between categories of solely sinus compression and indeterminate thrombosis/compression.

#### Timing of venography

*(Q3.3 In TBI patients, at what time should venography be performed to detect CVST?)*

Regarding the timing of venography, no direct evidence to inform the optimal timing of venography was identified. Sporadic reports that CVST can also develop with a delay after TBI were identified. Rischall et al. performed follow-up CT venography within 30 days post-trauma in 28/107 patients and noted 2/28 patients with delayed venous thrombosis (26).

### Therapeutics (topic 4)

The reported therapeutic management of CVST in TBI patients in the included studies was noted to be varying. The majority of studies however, especially studies of adult/all-age series, reported to treat at least a subset of the patients with CVST and TBI with anticoagulation (AC). The AC treatment regimens reported in the included studies were noted to be varying with regard to type of AC, timing of initiation, dose and treatment duration.

#### Safety of anticoagulation treatment

*(Q4.1 In TBI patients with CVST, is treatment with anticoagulation safe?)*

Four adult/all-age studies (23–25, 27) and one pediatric study (20) reported complication rates from anticoagulation (AC) treatment of CVST patients with TBI.

In a recently published study, Kim et al. systematically compared a group of adult CVST patients treated with AC to a group of adult CVST patients not treated with AC. In this retrospective single-center cohort design (25), 82/137 patients were treated with AC. Unfractionated heparin (UFH) was typically used in their practice in the acute phase, and initiation of treatment was withheld until interval CT scan showed stable traumatic hemorrhages ≥ 72 h after injury. Mean time to initiation was 6.57 ± 1.08 days post injury and mean AC treatment duration was 118 ± 20.47 days. A total of 9% (7/79) of AC treated patients developed complications in the form of new (*n* = 5) or worsening (*n* = 2) hemorrhage, with one mortality occurring among the patients with new or worsening hemorrhage. However, when comparing in-hospital mortality between the two groups, they found that the AC treated group had significantly lower in-hospital mortality compared to the group not treated with AC, despite these reported bleeding complications. Regarding predictors for developing bleeding complications, increasing time to AC treatment initiation was found to be significantly associated with reduced odds of developing bleeding complications (OR 0.63, 95% CI 0.42–0.94, *p* = 0.023).

In another series of 38 adults with CVST, Hersh et al. reported complication rates from the AC treatment of 22 of these patients (27). Type of AC, dosage and time of initiation were not specified. 3/22 were reported to have experienced minor complications (e.g., GI bleeding) and 2/33 were reported to have developed new or worsening intracranial hemorrhage resulting in one mortality. An additional two studies reported complication rates from AC treatment of a subset of their study-populations (23, 24). In both studies, four patients with CVSP and TBI were treated with AC and both studies reported no recorded complications from AC treatment.

In the single available pediatric study, Xavier et al. reported the complication rates from AC treatment of 14/20 children with CVST and TBI (20). Of these 14 children, 13 had concurrent intracranial hemorrhage. AC of varying type (UFH/LMWH/warfarin) was initiated at a median of 7 days (range 2–48 days) post-trauma after persistence (9/14) or propagation (5/14) of thrombus had been documented on venography. Three children were reported to have had minor bleeding complications (one epistaxis; two asymptomatic extension of hemorrhage) and further AC treatment was withheld. No children were reported to have experienced significant worsening of intracranial hemorrhage.

#### Anticoagulation and effect on outcome

*(Q4.2 In TBI patients with CVST, does treatment with anticoagulation improve outcome?)*

A single study was identified comparing outcome measures between a group of AC-treated patients with CVST and TBI and a group of CVST and TBI not treated by AC (25).

In their retrospective single center cohort design, Kim et al. included adult TBI patients with venography confirmed CVST (25). There were no significant differences between groups with regard to initial GCS, ISS or rates of different traumatic intracranial hemorrhages or neurosurgical interventions. When comparing in-hospital mortality between the groups, a significantly lower in-hospital mortality rate was found in the AC-treated group compared to the group not treated with AC [1% (1/82) in AC-group vs. 15% (8/55) in no-AC group, *p* = 0.009]. In logistic regression analysis assessing age, GCS, ISS, depressed skull fracture, intraparenchymal hemorrhage, AC treatment, craniotomy/craniectomy, external ventricular drain/intracranial pressure monitor as predictors of mortality, they found AC treatment to be a negative predictor of in-hospital mortality (OR 0.01 95 %CI 7.22 E-5–0.071).

Additionally, recanalization rates compared between groups of patients with AC treatment and without AC treatment were available from the study by Kim et al. and two additional smaller all-age studies (5, 23, 25).

Kim et al. found a significant difference between complete recanalization rates in their AC treatment group and no AC treatment group (54 vs. 32%, *p* = 0.012), but it should be noted that the AC treated group had a more than 3-fold longer mean time to last follow-up venography, which may have confounded this result (25).

In a series of 22 patients with CVST and follow-up venography by Almandoz et al., 5/22 patients were treated with AC while 17/22 of patients were not treated (5). Among the patients treated with AC, 60% (3/5) were found to have resolution of thrombus, 40% (2/5) had stable thrombus and none were found to have expansion of thrombus on follow-up imaging. In comparison, among patients not treated with AC, 41% (17/22) showed resolution of thrombus, 35% (6/17) had stable thrombus and 24% (4/17) had expansion of thrombus on follow-up imaging. Time to venography follow-up was not reported for the AC-treated vs. the non-AC-treated patients.

Grangeon et al. reported results from a series of five patients with CVST (23). Four out of five patients were treated with AC, three out of these four patients had follow-up venography and all of these venographies were reported to show complete resolution of thrombus. A single patient was not treated with AC and this patient was noted to have extension of thrombus.

#### AC treatment indications and treatment regimens

*(Q4.3: In TBI patients with CVST, what should be the indications for anticoagulation?)*

*(Q4.4: In TBI patients with CVST, what type of anticoagulation, and what dose, should be used?)*

*(Q4.5: In TBI patients with CVST, when should anticoagulation be started?)*

*(Q4.6: In TBI patients with CVST, what is the appropriate duration of anticoagulation?)*

Regarding indications for AC treatment, no specific evidence comparing different AC treatment indication protocols for CVST in TBI was available. The majority of studies, especially adult/all-age series, reported to treat at least a subset of patients with AC.

Regarding treatment regimens, no specific evidence comparing different types or doses of anticoagulation for CVST in TBI was identified. Variation with regards to type of AC used between the studies was noted.

Regarding timing of initiation and duration of treatment, no specific evidence was identified comparing different times of initiation or durations of treatment with AC. It was noted that Kim et al. found increasing time to AC treatment initiation to be significantly associated with reduced odds of developing bleeding complications (OR 0.63, 95% CI 0.42–0.94, *p* = 0.023) (25).

### Recanalization and follow-up (topic 5)

#### Recanalization rate

*(Q5.1: In TBI patients with CVST, what is the rate of recanalization of the affected venous sinus?)*

No studies systematically reporting recanalization rates at uniform times after trauma or diagnosis were identified. In addition, variability in the definition of CVSP used, proportion of patients with follow-up imaging (39–100%), and varying treatment of CVSP within the series precluded meaningful meta-analysis of results. Results are summarized in Table 5.

**Table 5:** Recanalization rates.

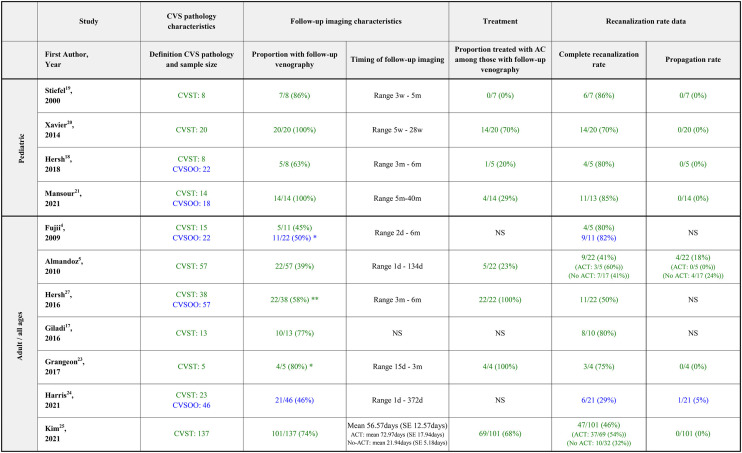

CVS, cerebral venous sinus; CVSOO, cerebral venous sinus outflow obstruction; CVST, cerebral venous sinus thrombosis; AC, anticoagulation; ACT, anticoagulation treatment; NS, not specified. Blue text color annotates CVSOO; Green text color annotates CVST.

^*^All surviving patients.

^**^AC treated fraction.

Seven adult/all age studies reporting recanalization rates of CVST were identified (4, 5, 17, 23–25, 27). In addition, Wang et al. reported recanalization rates in patients treated by an unconventional management algorithm which included intravenous thrombolytics (28). In the seven mentioned studies, the complete recanalization rates ranged between 41 and 80%, while the proportions of patients with propagation of thrombus ranged between 0 and 18%. The proportion of patients with CVST with follow-up imaging ranged between 39 and 80%. The proportion of patients treated with AC among those with follow-up imaging was reported in 4/7 series and ranged between 23 and 100%.

Four pediatric studies reported recanalization rates of CVST (18–21). The complete recanalization rates ranged between 70 and 86%. None of the studies reported findings of thrombus propagation at follow-up in any of the patients. Proportion of patients with CVST with follow-up imaging ranged between 63 and 100%. The proportion of patients treated with AC among those with follow-up in the four studies ranged between 0 and 70%.

#### Timing of recanalization

*(Q5.2: In TBI patients with CVST, when does recanalization typically occur?)*

Regarding the timing of recanalization, no systematic data determining the timing of when recanalization occurred was identified.

#### Follow-up

*(Q5.3: In TBI patients with CVST, should routine follow-up venography be performed?)*

*(Q5.4: In TBI patients with CVST, when should follow-up venography be considered?)*

No studies systematically reporting the yield of follow-up venography at set times after diagnosis of CSVT in TBI was identified. Furthermore, no data to directly informing the optimum timing of follow-up venography was identified.

## Discussion

Even though some evidence has emerged, especially in the last decade, the current evidence base is still limited when it comes to CVST in the setting of TBI.

Summarized from the included observational studies, venous infarctions were reported in 7–18% of patients in the adult/all age series and 38% of patients in a single pediatric series of CVST in TBI. In-hospital mortality was reported in 5–50% of patients with CVSP in the adult/all age series, while none of the pediatric series reported mortality in a total of 50 patients with CVST and TBI. One large registry-based study found an increased in-hospital mortality associated with CVSP and TBI compared to a control group with TBI alone in adjusted analysis. Furthermore, the same large registry-based study and another case-control study, found an increased mortality associated with midline CVSP and TBI as compared to TBI alone in adjusted analyses. When it comes to the diagnostics, no direct data to inform the appropriate indications for venography or the optimal diagnostic modality was available. Regarding treatment, a small number of studies report on the safety of AC treatment in CVST and TBI. In the largest and most recent of these, treatment with AC was associated with a significantly lower risk of in-hospital mortality, despite a small risk of new or worsening intracranial hemorrhage. When it comes to the optimal timing of initiation, dosage and duration of AC treatment, no comparative data was available. Complete recanalization rates were reported to range between 41 and 80% in adult/all age series and between 70 and 86% in pediatric series. The reported proportions of patients with propagation of thrombus ranged between 0 and 18% in adult/all age series, while none of the pediatric series reported findings of propagation of thrombus at follow-up in any of the patients. Data on recanalization rates was limited by the lack of studies with standardized timing of follow-up, and good data on the timing of recanalization, and hence optimal timing of follow-up venography, was unavailable.

The studies identified by this review, and therefore the review itself, is limited by the studies all being observational in nature, and many being small in sample size, and future studies are needed to increase knowledge on this condition in the setting of TBI. Meanwhile, the signals given by the current literature that CVST in TBI is associated with complications and increased risk of mortality indicate that management of the condition should be considered.

European stroke organization (ESO) guidelines provide recommendations for the management of CVST in general (12). However, the subgroup of traumatic CVST most importantly differs from spontaneous CVST in that concurrent traumatic intracranial hemorrhage often is present. This dictates a need to balance risk of extension of thrombus against risk of expansion of any traumatic hemorrhages on a case to case basis. Nonetheless, some aspects of ESO guideline recommendations may still be considered in the setting of TBI. Furthermore, and although still scarce, some evidence from the currently reviewed literature is useful for informing management of CVST in the setting of TBI.

### Management considerations

For the diagnostic management of CVST in TBI, fracture and/or epidural hematoma overlying a dural venous sinus have been reasonably well-established to be risk factors for its development. They should therefore, together with clinical suspicion, be indications for performing venography. Regarding diagnostic modality, ESO guidelines consider either CT or MR venography as options. In the setting of TBI, CT venography may be considered as the preferred modality due to its feasibility, especially in the acute phase, while MRI may be preferred for select cases where circumstances allow for MRI, especially in pediatrics to avoid radiation. Clear distinction between intramural thrombus (CVST) and external compression should be sought, both in future studies, but also in clinical practice, as it is likely to dictate different needs of treatment.

For the therapeutic management of CVST, ESO guidelines recommend LMWH in the acute phase. In our opinion this is a reasonable first option also in the setting of TBI, while UFH represents an alternative. The need to balance risk of thrombus extension against risk of expansion of any concurrent traumatic intracranial hemorrhages do however dictate a need to tailor the timing of initiation and dosage on a case to case basis. Limited data from the study by Kim et al. support that risk of hemorrhage is higher with earlier initiation of AC treatment. Furthermore, type and extent of traumatic hemorrhages is likely to affect the risk of AC treatment. Interval CT scans can be used to monitor progression of intracranial hemorrhage. We suggest initiation of LMWH after interval scan has documented relative stability of traumatic intracranial hemorrhages. In general, starting with a reduced dose and gradually increasing dosage over the next days to subtherapeutic/therapeutic levels under surveillance of repeat CT scans to monitor stability of traumatic intracranial hemorrhages should be considered. The timing of initiation, initiation dose, rate of dose increase and final dose should be individualized and related to the perceived risk of progression of traumatic intracranial hemorrhages.

The limited evidence suggesting that midline CVST may be associated with higher rates of complications and mortality suggests that the indication for AC treatment may be considered stronger in these instances.

In the subacute phase, ESO guidelines recommend oral anticoagulants in the form of warfarin. Since the publication of these guidelines, evidence has emerged showing direct oral anticoagulants (DOAC) to be non-inferior to warfarin both with regards to efficacy and safety in spontaneous CVST in both smaller RCTs [RESPECT-CVT (29); EINSTEIN-Jr (30)] and larger observational studies [ACTION-CVT (31)], and can therefore be considered an alternative. Switching to oral anticoagulation can be considered in the subacute phase of TBI if risk of progression and/or new hemorrhage is considered low. If continued subtherapeutic doses are deemed necessary, continued LMWH may be considered as an option.

Regarding the duration of treatment, ESO guidelines recommend treating with anticoagulation for 3–12 months. Compared to spontaneous CVST, where patients are more likely to have predispositions for venous thromboembolism, traumatic CVST is typically caused by a transient mechanical cause. Therefore, risk of recurrence is likely lower and management in the acute phase to prevent extension of thrombus is likely the most important. An initially planned treatment duration of 3 months can on this basis be considered adequate, but should be adjusted according to recanalization on follow-up venography.

Recanalization data from CVST in the setting of TBI is limited and at present unavailable to inform the optimal timing of follow-up. With treatment, follow-up schedules should consider the planned treatment duration. With an initial treatment duration of 3 months, a reasonable option could be to perform follow-up CT venography 1–2 weeks post trauma (before discharge from hospital) and 3 months post trauma. If CTV at 1–2 weeks show complete recanalization, AC treatment cessation may be considered. If CTV at 3 months shows a degree of recanalization, and given a mechanical cause of CVST (overlying fracture and/or EDH), AC treatment cessation may be considered. If CTV at 3 months shows no degree of recanalization, prolongation of AC treatment until a repeat venography at 6 months post trauma should be considered.

Whether the effects on outcome of CVST in TBI patients differ between pediatric and adult age groups remains undetermined. In general, there is however more available evidence to suggest that CVST is associated with complications and especially an effect on mortality in adult TBI patients compared to pediatric TBI patients. Additionally, there is also more available data on the safety of ACT in adult patients compared to pediatric patients with CVST and TBI. Hence, the foundation may be considered stronger for treatment of CVST in adult compared to pediatric TBI patients.

## Conclusion

Currently, the available evidence is insufficient for evidence-based treatment of CVST in the setting of TBI. However, there are clear indications in the presently available literature that CVST in TBI is associated with complications and increased mortality. Further studies are needed to confirm this and to provide evidence to support management decisions. These studies should be based on clear definitions of type of venous pathology with a clear distinction between intramural thrombosis and external compression.

## Data availability statement

The original contributions presented in the study are included in the article/Supplementary material, further inquiries can be directed to the corresponding author.

## Author contributions

DFN: conceptualization, methodology, investigation, writing—original draft, writing—review and editing, and project administration. ECS, MM-E, MA, and DAS: investigation and writing—review and editing. EJ: methodology, investigation, and writing—review and editing. EH: conceptualization, investigation, and writing—review and editing. TB: methodology, investigation, writing—review and editing, and project administration. All authors contributed to the article and approved the submitted version.
