# Supplementary material for: Cerebral venous sinus thrombosis in traumatic brain injury: A systematic review of its complications, effect on mortality, diagnostic and therapeutic management, and follow-up
Source: Front Neurol. 2023 Jan 9;13:1079579. doi: 10.3389/fneur.2022.1079579 (PMC9869151; doi:10.3389/fneur.2022.1079579)
Supplement: Supplementary file 1 [file Data_Sheet_1.PDF]

# REVIEWER COMMENT RESPONSES

**Title of the manuscript:** Cerebral venous sinus thrombosis in traumatic brain injury: a systematic review of its complications, effect on mortality, diagnostic and therapeutic management and follow-up.

**Manuscript Id:** 1079579

**REVIEWER 1:**

“The manuscript is very well written and adresses many of the aspects and complications associated with CVST in the settting of neurotrauma. Although the systematic review shows variable results the highlight results provided are significant.
I have two small comments:

**Comment 1:**
“regarding the duration of anticoagulation treatment - page 16 lines 56-74: I would tone down the recommendation to cease anticoagulation after 3 months. Although triggered by the neurotrauma, patient may have an underlying procoagulable condition such as APLA that would mandate a longer duration of anticoag treatment. Moreover, reaching certain recanalization goals may also guide us through the period required for anticoagulation treatment.”
**Authors’ response:**
Thank you for this comment. We agree that, although triggered by trauma, some patients may have an underlying predisposition for thrombus formation. We have toned down the recommendation to cease anticoagulation after 3 months accordingly.
**Changes to text:**
Discussion section, subheading “Management considerations”, paragraph four and five, lines 582-597: Please see various changes to the text.

**Comment 2:**
“A second comment - in some neurosurgical circumstances CSF leak may bring about intracranial hypotension as an additional underlying cause for CSVT. Taking the CSF leak into account and trying to ease the intracranial hypotension might be included in the course of treatment of such patients. Please add to the dicussion.”
**Authors’ response:**
CVST has been reported as a rare complication in idiopathic intracranial hypotension (eg Zhang et al., Cerebral Venous Thrombosis in Spontaneous Intracranial Hypotension: A Report on 4 Cases and a Review of the Literature. Headache. 2018;58(8):1244-1255 and Ferrante et al., Cerebral venous thrombosis in spontaneous intracranial hypotension: A report of 8 cases and review of the literature. J Neurol Sci. 2021;425:117467.) Zhang et al hypothesize that this increased risk is related to 3 factors: compensatory engorgement of the venous sinuses according to the Monroe-Kellie doctrine, sagging of the brain causing a mechanical effect on the venous sinuses, and loss of CSF leading to reduced CSF absorption into the venous sinuses and hence more viscous contents. IIH is a condition which is a condition usually understood as a more chronic condition and usually associated with spinal CSF leaks. We were unable to find any systematic literature (some case reports)

suggesting an association between CSF leak in the acute phase after TBI and CVST. In our opinion, CSF leaks typically associated with acute TBI differ from the situation seen with IIH as described above in several aspects. CSF leaks are often cranial, even though CSF leaks can occur with concurrent spinal injuries, seldom though do they cause hypotension in the acute phase. With cranial CSF leaks, it is quite uncommon to see a situation with intracranial hypotension in the acute phase after TBI, many patients with CSF leaks in the acute phase will also have intracranial hemorrhages/edema etc counteracting. We agree that this is a very interesting possible association, at the same time we feel the literature indicating the association between an acute CSF leak after TBI and CSVT is lacking. We further agree that CSF leaks in this phase should be treated if they do not subside spontaneously to eg prevent infection, and this is already part of standard practice in our institutions.

**Changes to text:**

No changes made.

**REVIEWER 2:**

**Comment 1:**

"I suggest the authors to **elaborate more about the mechanism of discussion** mentioned in line 155-156 that stated, '… Any disagreements on eligibility were settled by discussion and consensus in the study group'.

**Authors' response:**

Thank you for this suggestion, we have attempted to clarify this be editing the paragraph.

**Changes to text:**

Methods section, "Eligibility criteria and screening process" subheading, second paragraph, line 153-156: "Two authors (DFN and TB) independently first screened titles and abstracts and then full-text articles for eligibility. Any discrepancies between the two authors (DFN and TB) were resolved by discussion, first between the two authors (DFN and TB), and if necessary by discussion among all authors of the review."

**Comment 2:**

In the results section, in Line 300-301, both studies reported 0% mortality rate of pediatric population. It is interesting fact since in line 251-252, 257-258, and 263-264, there was tendency increased risk of mortality due to CVSP in adults. Is there any references that report any risk factors that may account? or any hypothesis **why mortality of pediatric population was 0%** ? Kindly add in the discussion.

"

**Authors' response:**

Thank you for commenting on this. We also found it interesting to note that no mortality was documented in the pediatric studies. The unadjusted mortality in the pediatric only studies among a total of 50 patients with CVST. The total sample size is quite small and derived from four small observational series. Only two of the four (the two smallest ones) series report TBI severity and none of the series provide functional outcome data for the pediatric patients with CVST as compared to a control group with TBI alone.

In our opinion, the effect of CVST on outcome should logically be related to the complications of CVST (venous infarction/ICH/focal and generalized oedema). Only one of the pediatric series reports on complications in the form of venous infarction (38%), none of the series report on any of the other complications from CVST mentioned. In our opinion, the data on

the pediatric population is too scarce to indicate if there is a real difference in effect on outcome between children and adults, and we have therefore been careful to emphasize this difference, but we have touched on this in the last paragraph of the discussion.

**Changes to text:**

Discussion, last paragraph, lines 599-600: "Whether the effects on outcome of CVST in TBI patients differ between pediatric and adult age groups remains undetermined. In general, there is however…".

**REVIEWER 3:**

"In this study Nettelman, et al evaluate the association of CVST with certain clinical questions in traumatic brain injury."

**Comment 1:**

"The questions of interest appear to be too broad and may be impossible to be adequately addressed in one study. For example in Table 1 – I am not certain that this study addresses or is able to answer most of the questions detailed in table 1. It may be helpful to either tailor the table to those questions that can or where truly answered in this study or to clearly state all questions which the authors found no studies that have addressed so that they can be areas of future studies. For example, this study does not address when it is safe to resume anticoagulation, what medication and what dose of anticoagulation is needed, even though these are some of the questions the authors aimed to systematically assess."

**Authors' response:**

Thank you for this comment. We agree that the key clinical questions are quite broad. When formulating the key clinical questions prior to conducting the systematic review, we chose these questions based on what was our review group thought were the most relevant key questions needing answers in today's clinical practice when treating patients with TBI. We were not surprised that the current literature is not able to provide evidence to answer all questions, and agree that many of the questions indeed cannot be answered well by the current evidence. At the same time, we also fully agree with your comment that this may be of value in itself in identifying areas/questions for future research. In the results section we have repeated each key clinical question and where there is no identified evidence to answer the question, this is stated following the question.

**Changes to text:**

No changes made

**Comment 2:**

Methods – Definition of the complications or presentation of interest are needed. Complications and/or clinical presentations of CVT go beyond venous infarction, (e.g seizures or status epilepticus may be a complication), and so a clear description of the complications of interest is required.

**Authors' response:**

Thank you for this comment. We agree that complications from CVST potentially can include different both clinical and radiological entities. We have now tried to clarify this in the methods section.

**Changes to text:**

Methods, first paragraph, line 126-128: “For the purpose of this review, complications from CVST was defined as venous infarction, intracerebral hemorrhage and focal or generalized edema secondary to CVST”.

**Comment 3:**
“Results - It may be helpful to modify table 2 to include some descriptive summary of the findings of included studies. on summarizing the major findings of interest, will give the reader a better quick summary of the context and findings of included studies.
**Authors’ response:**
Thank you for this comment. We agree that short written summaries can be helpful and did indeed formulate short written summaries of all included studies. We considered including this in the tables, but this resulted in very large tables covering several pages, and for this reason we considered readability to be compromised.
**Changes to text:**
No changes made

**Comment 4:**
“Some of the columns (in table 2), particularly those beginning from “n CVS ….” all seem very redundant, confusing and difficult to understand. Will recommend to just have a column reporting on the sample size of included studies and to take out all of the additional rows. Will suggest to replace the content of the columns with dots indicators with content that gives the reader an overview of the study findings.“
**Authors’ response:**
We agree that the current setup in table 2 with many columns dedicated to the type and number of cerebral venous sinus (CVS) pathology is a bit two detailed and that it is difficult to read. We have now revised the setup with fewer columns dedicated to this purpose. Still, we think it is important to keep the subdivisions of type of CVS pathology used in the different studies, because it illustrates the potential difficulty in direct comparison of studies and the need to use clear definitions in future studies. As a result of fewer columns dedicated to this purpose, more of the total content is now with dot indicators as a result.
**Changes to tables:**
-Columns and setup revised in table 2.

**Comment 5:**
“In the results section, it may be helpful to organize the results in a somewhat chronological order beginning with diagnosis, treatments, complications and ending in in-hospital mortality.”
**Authors’ response:**
Thank you for this comment. The order of the topics, after which both the order of the key clinical questions and the results section is organized, is something we have discussed in the review group and we agree that a more traditional chronological order would be an alternative. The current order was chosen because we believe that the complications from CVST and its effect on mortality in TBI patients form a basis on which the diagnostic and therapeutic management of the condition should be considered. We have described this reasoning in the methods section, first paragraph, line 124-126.
**Changes to text:**
No changes made.

**Comment 6:**
“Table 2. Will suggest to take away the column on age groups since it is already in column 1 and to have an additional subdivision of column 1 for all ages into adults separately and all ages.“
**Authors’ response:**
Thank you for this comment, we have as suggested removed the column on age group.
**Changes to table:**
Column on age groups removed in table 4.

**Comment 7:**
“In the discussion, a paragraph focused on the limitations of the study is required. E.g most of the included studies were small single center studies, all were retrospective, etc.”
**Authors’ response:**
Thank you for this comment. We have made changes to the second paragraph in the discussion accordingly.
**Changes to text:**
Discussion, second paragraph, line 524-525: “The studies identified by this review, and therefore the review itself, is limited by the included studies all being observational in nature, and many being small in sample, and…”

**Comment 8:**
“Table 4- CSVT used interchangeably with CVST, revise as required.”
**Authors’ response:**
Thank you for pointing this out, the spelling is now corrected in the tables.
**Changes to table:**
Misspelled abbreviations (CSVT) corrected in tables.
